# On the Capabilities of Transition Metal Carbides for Carbon Capture and Utilization Technologies

**DOI:** 10.1021/acsami.4c03735

**Published:** 2024-05-24

**Authors:** Hector Prats, Arturo Pajares, Francesc Viñes, Pilar Ramírez de la Piscina, Ramon Sayós, Narcís Homs, Francesc Illas

**Affiliations:** † Department of Chemistry, Physical and Theoretical Chemistry Laboratory, University of Oxford, South Parks Road, Oxford OX1 3QZ, U.K.; ‡ Sustainable Materials, Flemish Institute for Technological Research (VITO NV), Boeretang 200, Mol 2400, Belgium; § Departament de Ciència de Materials i Química Física & Institut de Química Teòrica i Computacional (IQTCUB), Universitat de Barcelona, Martí i Franquès 1-11, Barcelona 08028, Spain; ∥ Departament de Química Inorgànica i Orgànica, Secció de Química Inorgànica and Institut de Nanociència i Nanotecnologia (IN2UB), Universitat de Barcelona, Martí i Franquès 1-11, Barcelona 08028, Spain; ⊥ Institut de Recerca en Energia de Catalunya (IREC), Jardins de les Dones de Negre 1, Barcelona 08930, Spain

**Keywords:** transition metal carbides, heterogeneous catalysis, CO_2_ utilization, infrared spectroscopy, C vacancies

## Abstract

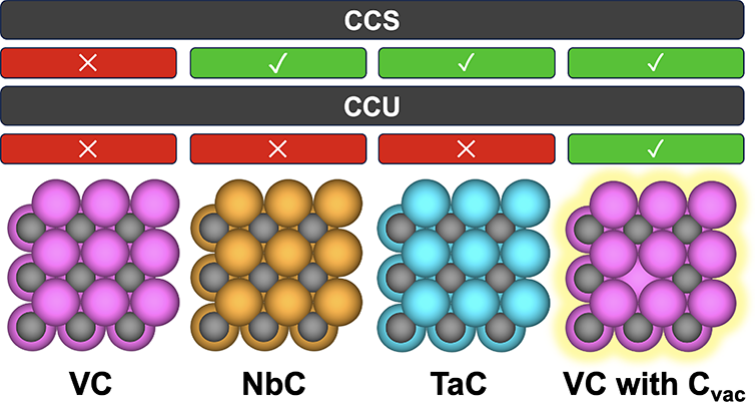

The search for cheap and active materials for the capture and activation of CO_2_ has led to many efforts aimed at developing new catalysts. In this context, earth-abundant transition metal carbides (TMCs) have emerged as promising candidates, garnering increased attention in recent decades due to their exceptional refractory properties and resistance to sintering, coking, and sulfur poisoning. In this work, we assess the use of Group 5 TMCs (VC, NbC, and TaC) as potential materials for carbon capture and sequestration/utilization technologies by combining experimental characterization techniques, first-principles-based multiscale modeling, vibrational analysis, and catalytic experiments. Our findings reveal that the stoichiometric phase of VC exhibits weak interactions with CO_2_, displaying an inability to adsorb or dissociate it. However, VC often exhibits the presence of surface carbon vacancies, leading to significant activation of CO_2_ at room temperature and facilitating its catalytic hydrogenation. In contrast, stoichiometric NbC and TaC phases exhibit stronger interactions with CO_2_, capable of adsorbing and even breaking of CO_2_ at low temperatures, particularly notable in the case of TaC. Nevertheless, NbC and TaC demonstrate poor catalytic performance for CO_2_ hydrogenation. This work suggests Group 5 TMCs as potential materials for CO_2_ abatement, emphasizes the importance of surface vacancies in enhancing catalytic activity and adsorption capability, and provides a reference for using the infrared spectra as a unique identifier to detect oxy-carbide phases or surface C vacancies within Group 5 TMCs.

## 1 Introduction

Nowadays, considerable research endeavors are devoted at developing or improving carbon dioxide (CO_2_) capture and conversion technologies,^1−3^ mostly fostered by the urgent need of reducing the concentration of CO_2_ in the atmosphere. These processes aim at mitigating the CO_2_ greenhouse effect and its concomitant effects on the climate change.^4^ Here, turning the CO_2_ economy into a waste-to-product economy model also appears as an opportunity. Initial efforts lead to the so-called carbon capture and storage (CCS) technologies, requiring to this end materials capable of adsorbing/absorbing significant amounts of CO_2_ at normal conditions of temperature and CO_2_ partial pressure ().^5,6^ The inherent chemical stability of a CO_2_ molecule complicates this pursuit, requiring materials capable of inducing the necessary charge transfer from the substrate to the adsorbed CO_2_ molecule to transform it into an activated, bent CO_2_^δ−^ species—a prerequisite for utilizing CO_2_ as a chemical feedstock in CO_2_ capture and utilization (CCU) technologies.^1−3^

First-principles studies based on density functional theory (DFT) proposed transition metal carbides (TMCs) as an appealing family of materials able to capture and activate CO_2_ even at stringent conditions of temperature, *T*, and .^7,8^ For some TMCs, such as ZrC and HfC, these conditions could imply temperatures as high as 200 °C and a gas source with a low CO_2_ content, such as atmospheric air, with a current CO_2_ average content of 40 Pa.^9^ Experimental validation of these theoretical predictions followed, with experiments on TiC, ZrC, and VC samples confirming the CO_2_ capture capabilities through a combination of X-ray photoemission spectroscopy (XPS) and DFT binding energy estimates.^10^

Further than that, the CO_2_ CCS was envisaged on two-dimensional (2D) TMCs even under more stringent conditions,^11,12^ a point also experimentally confirmed by selective chemical resolution of CO_2_ over N_2_.^13^ Even though the CCS capabilities of TMCs are proven, an open question that still needs to be answered is whether such materials are also adequate for CCU technologies *per se*. This is, are such TMCs able to catalyze the CO_2_ conversion? For instance, the CO_2_ decomposition into CO + O is considered as a textbook example. Would this require some sort of assistance? Or, on the contrary, would the TMCs usage be restricted only to CCS technologies?

To answer these questions, we evaluate here the CCS and possible CCU capabilities of Group 5 TMCs (VC, NbC, and TaC) by combining experimental tests with multiscale modeling from first principles. As shown below, calorimetric CO_2_ adsorption measurements align well with theoretical estimates, while the combined analysis of experimental and simulated infrared (IR) spectra unveils potential surface oxidation, leading to the formation of oxy-carbide phases—a well-documented occurrence in TMCs exposed to molecular oxygen.^14^ The impact of surface carbon vacancies on CO_2_ adsorption and the TMC catalytic behavior is also explored, a phenomenon encountered in the case of VC.^15^ Finally, our investigation delves into the configuration of the TMC adlayer across a diverse range of temperatures and CO_2_ partial pressure conditions, employing kinetic modeling simulations that consider the effects of diffusion and lateral interactions. This work provides valuable insights into the distinctive CO_2_ interaction mechanisms of Group 5 TMCs, shedding light on their potential applications in CCS and CCU technologies while emphasizing the importance of surface vacancies in enhancing their catalytic activity. Moreover, it provides a practical guide to using the IR spectrum as a fingerprint to detect the presence of oxy-carbide phases or surface carbon vacances in Group 5 TMCs.

## 2 Methodology

### 2.1 Preparation of Samples

All Group 5 TMC samples were prepared based on a sol–gel method as previously reported.^15^ In short, alcoholic solutions of VO(isopropoxide)_3_ (Alfa Aesar, 96%), VOCl_3_ (Alfa Aesar, 99%), Nb(OC_2_H_5_)_5_ (Alfa Aesar, 99.95%), or TaCl_5_ (Alfa Aesar, 99.6%) were prepared under Ar. Ethanol was used for VC and NbC preparation; however, due to low solubility of TaCl_5_ in ethanol, methanol was used for TaC preparation. For VC, two samples, VC-Pr and VC-Cla, were prepared by using VO(isopropoxide)_3_ and VOCl_3_ as vanadium precursors, respectively. For the preparation of these two VC samples, 4,5-dicyanoimidazole (Manchester Organics, 96%) was added to the alcoholic solutions as a carbon source. The solution was then stirred until ethanol evaporated resulting in a gel, and the latter was thermally treated under Ar flow at 1373 K for 5 h. In a prior study,^15^ we observed the prevalent presence of the V_8_C_7_ phase in the vanadium carbide sample prepared using VO(isopropoxide)_3_ (VC-Pr). In contrast, the sample prepared using VOCl_3_ (VC-Cla) exhibited a higher presence of the stoichiometric VC phase. Additionally, VC-Cla was heated up until 1623 K, resulting in a new sample named VC-Clb with a higher presence of stochiometric VC, with the aim to analyze the impact of C vacancies. NbC and TaC samples were prepared following the procedure described above but treated under Ar flow at 1473 K for 5 h. After the thermal treatment, samples were contacted with air without previous passivation. The amount of the metal and C precursors added for the preparation of the different samples can be found in Section S1 of the Supporting Information (SI).

### 2.2 Characterization

Powder X-ray diffraction (XRD) measurements were conducted in the range 2θ = 4–100°, with a step size of 0.017° and an acquisition time of 80 s per step. The measurements were performed using a PANaltycal X′Pert PRO MPD Alpha1 powder diffractometer, utilizing a Ge(111) primary monochromator and Cu Kα1 radiation source (λ = 1.5406 Å). The average crystallite size of the transition metal carbide phases was determined using the Debye–Scherrer equation. The specific surface area (*S*_BET_) was assessed by conducting multipoint BET analysis on the N_2_ adsorption isotherms. N_2_ adsorption–desorption isotherms were measured at 77 K by using a Micromeritics Tristar II 3020 instrument. Additionally, the pore size distribution was determined using the BJH (Barret–Joyner–Halenda) method. Scanning electron microscopy (SEM) images were obtained with a ZEISS Auriga instrument operating at an accelerating voltage of up to 20 keV. Transmission electron microscopy (TEM) images were collected employing a JEOL J2010F microscope operated at an accelerating voltage of up to 200 kV. Raman spectra were acquired using a Jobin-Yvon LabRam HR800 spectrometer coupled with an Olympus BXFM microscope, employing a 532 nm laser and a CCD detector. To minimize laser-induced heating effects during data collection, the laser power was restricted to 0.75 mW.

For the H_2_-temperature-programmed reduction (H_2_-TPR) experiments, a Micromeritics AutoChem II 2920 chemisorption instrument was utilized. The samples underwent pretreatment at 363 K under a He atmosphere before being subjected to H_2_/Ar (12% v/v) flow. The temperature was then increased to 1073 K at a rate of 10 K min^–1^.

The adsorption enthalpy of CO_2_ onto the different samples was measured using a Sensys evo TG-DSC instrument from Setaram, equipped with a 3D thermal flow sensor and was coupled online to a ThermoStar GSD320T1 mass spectrometer analyzer. The sample (100 mg) was first dried under Ar flow (50 mL min^–1^) at a heating rate of 10 K min^–1^ up to 353 K for 30 min. Subsequently, the sample was heated to 823 at 10 K min^–1^ under H_2_/Ar (10% v/v) for 1 h to remove impurities and/or surface oxy-carbide species and then cooled to 303 K under Ar flow. After that, a mixture of CO_2_/He (10% v/v) was introduced to the sample at 303 K with a flow rate of 10 mL min^–1^ until no further changes in mass or heat flow were detected. The exothermic peaks corresponding to the adsorption of CO_2_ were integrated to determine the total enthalpy of the adsorption. Additionally, the mean adsorption energy for CO_2_ was calculated based on the total amount of CO_2_ adsorbed during the experiment.

On the other hand, CO_2_, CO, and CO_2_/H_2_ (1/3 mol/mol) adsorption experiments were conducted using *in situ* diffuse reflectance infrared spectroscopy (DRIFTS) with a Bruker VERTEX 70 FTIR spectrometer equipped with an MCT detector. This setup included a Harrick Scientific HVCDRP-4 catalytic chamber and was coupled online to a ThermoStar GSD320T1 mass spectrometer analyzer. During the experiment, the spectra were acquired by averaging 256 scans at a spectral resolution of 4 cm^–1^. Approximately 30 mg of sample underwent *in situ* treatment within the DRIFTS cell under He flow (20 mL min^–1^) up to 573 K for 30 min. The gas flow was then switched to H_2_ for 1 h before cooling the sample to 308 K under He, with the corresponding background spectrum registered. Afterward, in separate experiments, gas mixtures of CO_2_/He (10% v/v), CO/He (10% v/v), or CO_2_ (10% v/v)/H_2_ (30% v/v)/He were introduced at 308 K with a flow rate of 20 mL min^–1^ and allowed to interact with the sample for 20 min. Then, the gas flow was switched to He, and the final spectra were recorded. In every case, the effluent was continuously analyzed by online mass spectrometry (MS). The *m*/*z* = 30 signal corresponding to CO was analyzed due to the overlapping of the CO_2_ signal at *m*/*z* = 28.

### 2.3 CO_2_ Reactivity and RWGS Catalytic Test

CO_2_ reactivity and reverse water gas shift (RWGS) reaction studies were performed using a tubular fixed bed reactor made of 316-L stainless steel, measuring 305 mm in length with an inner diameter of 9 mm. A thermocouple was in direct contact with the sample within the reactor setup. Then, 150 mg of sample was diluted within SiC up to 1 mL. For CO_2_ reactivity studies, the sample was heated to 873 K under N_2_, and then, a flow of CO_2_/Ar (20%v/v) (50 mL min^–1^) was introduced for 140 min.

For the catalytic studies of the RWGS reaction, the sample was initially heated to 573 K under N_2_. Subsequently, a reactant mixture of CO_2_/H_2_/N_2_ = 1/3/1 was introduced into the system with a gas hourly space velocity (GHSV) of 3000 h^–1^. The catalytic behavior of the samples in the RWGS was investigated over a temperature range from 573 to 873 K at a pressure of 1 bar. At each temperature point, the conversion and product distribution were determined by averaging results from at least four separate analyses with each analysis conducted over a period of 2 h at the specified temperature. The CO_2_ conversion () and the selectivity toward a specific product *i* were defined and calculated as follows:
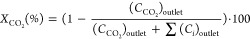
1
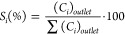
2where (*C*_*i*_) and  are the molar concentrations of the *i* product (CO or CH_4_), respectively. The analysis and quantification of products for both the CO_2_ reactivity and RWGS reaction studies were performed using a Varian 450-GC (Gas Chromatograph) instrument equipped with a thermal conductivity detector (TCD). This instrumentation allowed for accurate measurement and identification of the reaction products.

### 2.4 Computational Methods

DFT calculations were performed using the Vienna *Ab Initio* Simulation Package (VASP) computational suite,^16^ using the Perdew–Burke–Ernzerhof (PBE)^17^ exchange–correlation (*xc*) functional, and including dispersion through the D3 method developed by Grimme to account for dispersion interactions.^18^ The effect of the core electrons on the valence electron density was described by the Projected Augmented Wave (PAW) method of Blöchl^19^ and later implemented in VASP by Kresse and Joubert,^20^ whereas the valence electron density was expanded in a plane wave basis set with a cutoff kinetic energy of 520 and 415 eV for bulk and surface calculations, respectively. For bulk structure relaxation, electronic and force convergence tolerances of 10^–6^ eV and 10^–3^ eV·Å^–1^, respectively, were imposed, and a dense **Γ**-centered **k**-point grid of 80/*a* × 80/*b* × 80/*c* was used, where *a*, *b*, and *c* are the lattice vectors. The slab models for the stoichiometric carbides (i.e., TMCs, TM = V, Nb, or Ta) were constructed from the optimized bulk structures. The slab models for the corresponding carbides with a surface C vacancy (i.e., TMC_1–*x*_) were constructed by removing a surface C atom from the TMC slab models and reoptimizing. Finally, the slab models for the corresponding oxycarbides (i.e., TMOC) were constructed by replacing all surface C atoms by O atoms.

To avoid interactions between the periodically repeated slab images, a vacuum width of at least 18 Å was added between the interleaved slabs and a dipole correction was applied along the vacuum direction. For the structure optimization of the clean slab models, the adsorbed species, and the calculation of the transition states (TS), electronic and force convergence tolerances of 10^–5^ eV and 10^–2^ eV·Å^–1^, respectively, were imposed, and a **Γ**-centered **k**-point grid of 60/*a* × 60/*b* × 1 was used. Note that when calculating the number of **k**-points in a specific direction, noninteger values were rounded up to the nearest integer. The formation energy of CO_2_, CO, and O species (*E*_*f*,*i*_) is calculated with respect to gas-phase CO_2_ and O_2_ molecules (see Section S3 in the Supporting Information). The simulated IR spectra were acquired from vibrational analysis, where the intensities are obtained as the square of the change of the dipole moment perpendicular to the surface associated by the vibrational frequency; details from this procedure are found elsewhere.^10,21,22^ The CatLearn’s Bayesian transition state search module (ML-NEB)^23^ was used to locate all the TS, and the vibrational frequencies of the TS were analyzed to check that only one imaginary mode is present. For crystal structure manipulations and data analysis, we used the Python Materials Genomics (pymatgen)^24^ and the Atomic Simulation Environment (ASE)^25^ Python libraries. The enthalpies and free energies for each species were computed using the ASE thermochemistry module^25^ using the ideal gas model for gas-phase molecules and the harmonic oscillator model for adsorbed species.

Kinetic simulations were carried out using the graph-theoretical kinetic Monte Carlo (kMC) approach^26^ combined with cluster expansion Hamiltonians^27,28^ for the surface energetics, as implemented in the ZACROS code.^26^ The kMC lattice model consists of a 10 × 10 periodic custom grid of two points—or a total of 200 points—representing surface sites, where the different species can adsorb, desorb, react, or diffuse. Two site types were used, namely, tM and tC, to describe top metal (i.e., V, Nb, or Ta) and top carbon sites. The reaction network involves a total set of four reversible reactions, namely, CO_2_ and O_2_ adsorption, CO_2_ dissociation, and O species diffusion (see Table S1 in the Supporting Information). The cluster expansion used in our model includes first-nearest-neighbors pairwise lateral interactions between all possible pairs among CO_2_, CO, and O (see Table S2 in the Supporting Information). The effect of lateral interactions in the energy barriers is modeled by using the Bro̷nsted–Evans–Polanyi (BEP) relations.^28^ The kMC input files were automatically generated using the ZacrosTools Python library.^29^

## 3 Results and Discussion

### 3.1 Characterization of As-Synthesized Carbides

**Figure 1:**
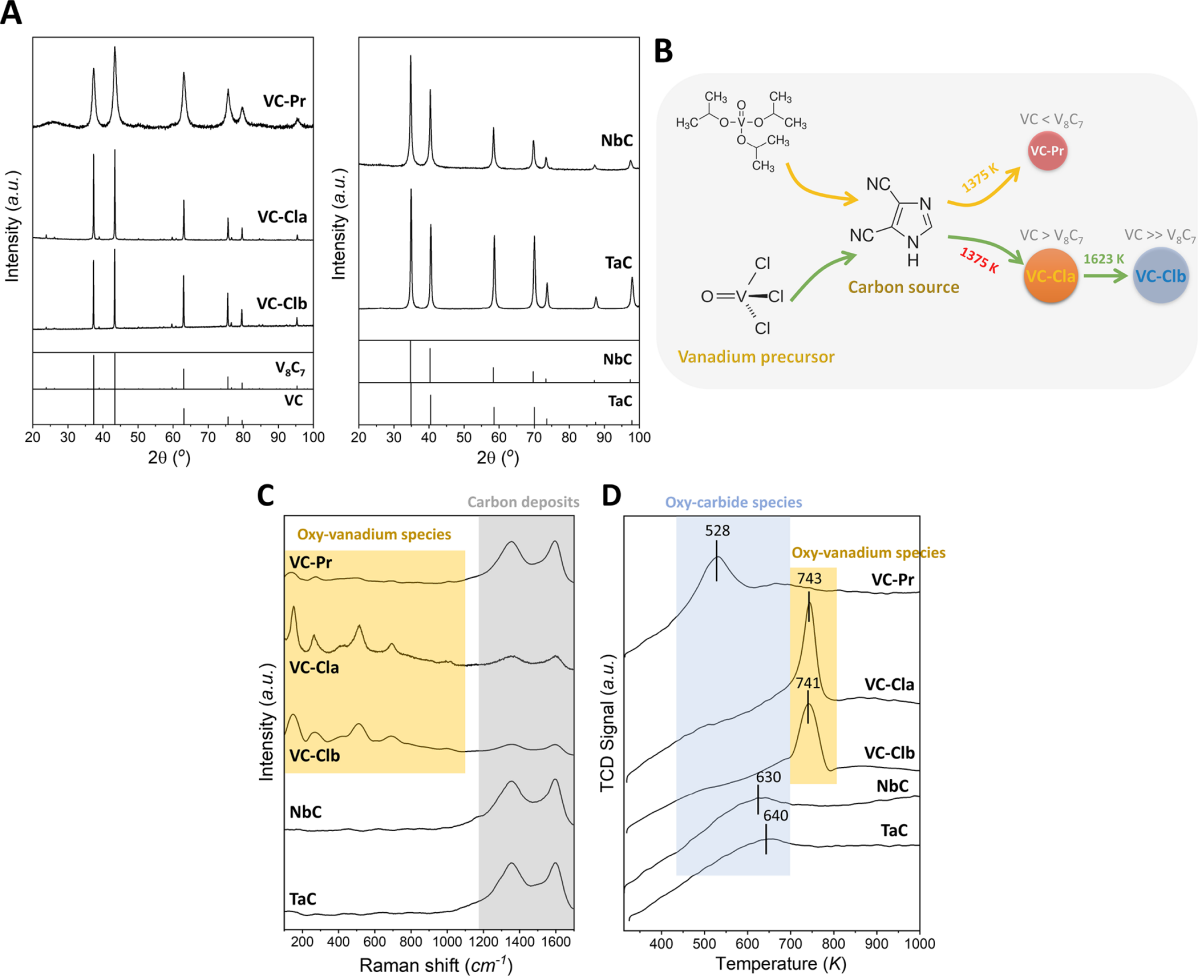
Characterization and preparation scheme of as-synthesized Group 5 TMCs: (A) XRD patterns, (B) preparation of the VC samples, (C) Raman spectra, and (D) H_2_-TPR profiles.

The XRD patterns of all five carbide samples are depicted in Figure 1A. For VC, characteristics peaks at 2θ = 37.4, 43.4, 63.0, 75.6, and 79.7°, corresponding to cubic V_8_C_7_ (JCPDS 35-0786) and/or VC (JCPDS 01-073-0476) phases are observed. Note that in the case of vanadium carbides, the substoichiometric V_8_C_7_ phase (1 C vacancy per 8 V atoms) is more stable than the stoichiometric VC phase.^30^ In a previous study, we investigated the presence of both stoichiometry VC and V_8_C_7_ phases in vanadium carbide samples according to the preparation method employed in this work (cf. Figure 1B).^15^ From PED-ASTAR analysis, we determined a prevalent presence of the stoichiometric VC phase in VC-Cla. On the other hand, for VC-Pr, a more accurate fitting indexation was achieved by assuming the presence of the V_8_C_7_ phase in most of the analyzed regions.^15^ Additionally, VC-Clb was obtained by heating VC-Cla to 1623 K. In this case, it can be assumed that a significant presence of VC phase exists in VC-Clb as the phase transition from V_8_C_7_ → VC is favored with an increase in temperature (cf. Figure 1B).^31^ Therefore, while all three VC samples contain C vacancies, the concentration of vacancies decreases in the following order: VC-Pr > VC-Cla > VC-Clb. For the NbC sample, the peaks at 2θ = 34.7, 40.3, 57.3, 69.7, 73.3, 87.1, and 97.4° are attributed to the cubic NbC phase (JCPDS 38-1364). Finally, for the TaC sample, peaks at 2θ = 34.9, 40.5, 58.5, 70.0, 73.6, 87.5, and 97.9° are assigned to the presence of the cubic TaC phase (JCPDS 35-0801). The perfect match of the experimental diffraction patterns with those of standard references, and the absence of additional peaks, points that the concentration of C vacancies in NbC and TaC samples is significantly lower than in the case of the VC samples. This agrees with the DFT-calculated vacancy formation energies, which predict surface C vacancies to be more stable in VC(001) than on either NbC(001) or TaC(001), as shown in Table S3 in the Supporting Information. On the other hand, no XRD peaks indicative of crystalline oxides were detected in any case.

Table 1 presents the crystallite sizes of the samples determined through the XRD analysis. The method used for vanadium carbide preparation influenced the resulting crystallite sizes. VC-Pr, prepared using VO(isopropoxide)_3_ as a vanadium precursor, exhibited the smallest crystallite size (10 nm). In contrast, VC-Cla, prepared by using VOCl_3_, showed a larger crystallite size of 64 nm. Heating VC-Cla to 1623 K (VC-Clb) resulted in a slight increase in the vanadium carbide crystallite size, consistent with the findings from TEM analysis (Figure S1). Both NbC and TaC samples demonstrated similar crystallite sizes of approximately 20 and 22 nm, respectively (Table 1).

**Table 1:** Crystallite Size (XRD), Amount of Adsorbed CO_2_, CO_2_ Adsorption Heat Values at 303 K, and CO Production of Samples at 873 K^d^

			**CO**_**2**_**adsorption heat (eV)**		**CO production**
**sample**	**crystallite size (nm)**	**adsorbed CO_2_** (μgCO_2_ mg_cat_^–1^)	**Exp.**	**Calc.**^a^	**(mmolCO kg**_**cat**_^**–1**^**)**^c^
VC-Pr	10	7.40	–0.54	–0.84^b^	2913
VC-Cla	64	5.94	–0.25	–0.15^a^	2112
VC-Clb	70	5.52	–0.22	–0.15^a^	1980
NbC	20	0.89	–1.02	–0.93	271
TaC	22	0.91	–0.94	–1.17	226

a VC(001).

b VC_1–*x*_(001).

c From CO_2_ reactivity studies.

d Calculated CO_2_ adsorption heats at 303 K have been computed using the ASE thermochemistry module (ref (25)).

All samples showed a rather uniform distribution of regular particles with no preferential morphology, as observed from SEM analysis (Figure S2). VC-Pr (315 m^2^g^–1^), VC-Cla (204 m^2^g^–1^), NbC (45 m^2^g^–1^), and TaC (51 m^2^g^–1^) are mesoporous materials, with an average pore size of 3.4–5.1 nm (Figure S3). All samples exhibited Raman bands with peaks centered at 1358 and 1595 cm^–1^ (Figure 1C), which are attributed to the presence of residual amorphous carbon resulting from the preparation process. Furthermore, for VC-Cla and VC-Clb, multiple bands were observed within the 100–1100 cm^–1^ range. These bands are associated with surface oxy-vanadium species, likely formed during exposure of the samples to air.^32,33^ The presence of a low intensity band at 991 cm^–1^ may suggest the existence of a very low amount of crystalline V_2_O_5_ in both cases.^34^ In contrast, VC-Pr displayed significantly lower intense Raman bands in the 100–1100 cm^–1^ range compared with VC-Cla and VC-Clb. This suggests a lower presence of surface oxy-vanadium species. NbC and TaC did not show Raman peaks corresponding to NbO_*x*_ and TaO_*x*_, respectively.^35,36^

H_2_-TPR experiments were conducted on all samples to confirm the presence of oxide species (cf. Figure 1D). In all cases, only very low amounts of hydrogen consumption were determined. The H_2_-TPR profiles of VC-Cla and VC-Clb revealed a H_2_ consumption peak at about 743 K of 0.77 and 0.70 mmol H_2_ g_cat_^–1^, respectively, attributed to the reduction of mono- or polymeric oxy-vanadium species. This is because the reduction of amorphous V_2_O_5_ takes place at about 852 K and that of crystalline V_2_O_5_ at higher temperatures.^34,37^ The minimal H_2_ consumption peaks observed for VC-Pr (0.41 mmol H_2_ g_cat_^–1^), NbC (0.16 mmol H_2_ g_cat_^–1^), and TaC (0.05 mmol H_2_ g_cat_^–1^), at temperature ranging from 528 to 640 K, can be associated with the reduction of oxy-carbide species, similar to what has been proposed for MoC_*x*_ catalysts.^38^ The more pronounced H_2_ consumption peak observed for VC-Cla and VC-Clb in comparison to VC-Pr correlates with the larger presence of oxy-vanadium species in VC-Cla and VC-Clb, as deduced from Raman spectroscopy analysis (cf. Figure 1C). Reductions of Nb_2_O_5_ and Ta_2_O_5_ are not observed within this temperature range as they take place at higher temperatures (>1173 K).^39^

### 3.2 Strength and Capability of CO_2_ Adsorption

Computed *E*_ads_ values for CO_2_ adsorption on the different TMCs show that, while CO_2_ interacts weakly with VC (−0.15 eV), the interaction with NbC and TaC is quite strong (−0.93 and −1.17 eV, respectively), as shown in Table 1. Thus, CO_2_ adsorption strength follows the trend VC < NbC ≈ TaC. However, VC can bind CO_2_ almost as strongly as NbC and TaC if C vacancies are present (*E*_ads_ on VC_1–*x*_ is −0.84 eV). The mechanism by which C vacancies enhance the CO_2_ adsorption is explained as follows. Unlike transition metals (TMs), the interaction between TMCs and reaction intermediates is highly influenced by electrostatic interactions. As described in a recent work,^40^ the preferred adsorption site does not follow simple valency rules (as opposed to TMs) but correlates with the atomic charge of surface atoms. This is because of the ionic nature of TMCs, where C atoms carry a negative charge and metal atoms carry a positive charge. In a C vacancy site, the surrounding metal atoms are more prone to interact with negatively charged atoms, such as the O atom from CO_2_. This creates a new adsorption mode for CO_2_ on the vacancy site where one of the two O atoms is directly linked to two neighboring metal atoms (Figure S6), which results in a stronger adsorption compared to the clean carbide.

**Figure 2:**
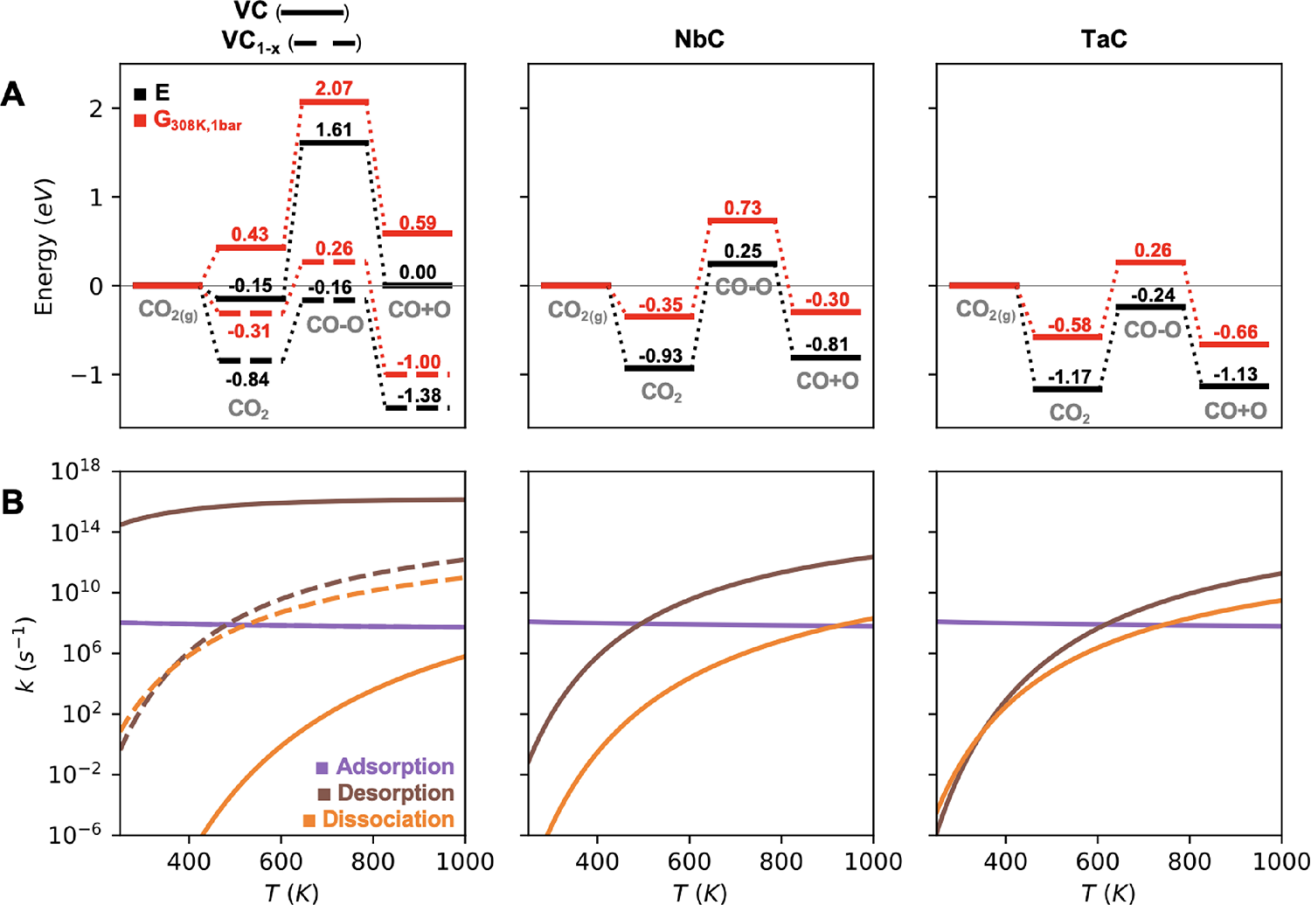
(A) Potential energy (black) and Gibbs free energy (red, *T* = 308 K,  = 1 bar) profiles for CO_2_ adsorption and dissociation on the (001) surfaces of VC and VC_1–*x*_, NbC, and TaC. The final states correspond to CO and O species at infinite separation (no lateral interaction). Gibbs free energies for gas-phase and adsorbed species have been computed by means of the ideal gas and harmonic oscillator models, respectively, as implemented in the ASE thermochemistry module (ref (25)). (B) Rate constants in the temperature range 300–1000 K for CO_2_ adsorption (purple,  = 1 bar), desorption (brown), and dissociation (orange) on the (001) surfaces of VC, NbC, and TaC. Rate constants have been computed from the partition functions as detailed in the Supporting Information of ref (26) .

The measured CO_2_ adsorption heats at 303 K for the different samples agree quite well with the computed DFT values (Table 1). Table 1 also shows the amount of adsorbed CO_2_. The highest amount of adsorbed CO_2_ and the strongest CO_2_ adsorption heat for the VC-Pr sample can be ascribed to a higher concentration of C vacancies compared to those of VC-Cla and VC-Clb. Moreover, the lower CO_2_ adsorption heat of VC-Clb compared with VC-Cla confirms the higher prevalence of the stoichiometric VC phase in VC-Clb. On the other hand, for NbC and TaC, the lower amount of adsorbed CO_2_ could be related with their lower surface area. Finally, note that the measured adsorption heats cannot be univocally attributed to CO_2_ as the CO and the O species might also be present. However, Figure 2A shows that the relative stability of adsorbed CO_2_ and CO + O species is very similar, meaning that the computed PBE-D3 values would also agree with the experimental results in the case that CO_2_ dissociates into CO + O. Moreover, as will be shown in Section 3.4, the kMC simulations for the three stoichiometric carbides predict that at 303 K, no CO and/or O species are present.

### 3.3 Thermodynamics of CO_2_ Activation

The computed potential energy and Gibbs free energy profiles in Figure 2A reveal that while CO_2_ dissociation on stoichiometric VC is prohibited (i.e., the potential energy barrier is 1.76 eV), it can occur on NbC and TaC, which exhibit energy barriers of 1.18 and 0.93 eV, respectively. The CO_2_ dissociation ability of VC, however, can be drastically improved when C vacancies are present because the new adsorption sites provided by such C vacancies can lower the energy barrier from 1.76 to only 0.68 eV. The computed free energy profiles show that, at 308 K and  = 1 bar, CO_2_ dissociation should be quite feasible on VC_1–*x*_ and TaC, both exhibiting a Gibbs free energy barrier of only 0.26 eV with respect to CO_2(g)_. For VC_1–*x*_, the dissociated products (CO + O) are significantly more stable than adsorbed CO_2_, while in TaC, the dissociation step is almost thermoneutral. For the case of NbC, CO_2_ dissociation is in principle feasible, although at a lower rate since the corresponding Gibbs free energy barrier is 0.73 eV. Finally, the free energy profile for stoichiometric VC suggests that, at these conditions, the surface would be clean as the free energy barrier is 2.07 eV and CO_2_ adsorption would not be favored.

### 3.4 Kinetics of CO_2_ activation

To further evaluate the possible molecular or dissociative chemisorption of CO_2_ on the studied TMCs at a wide range of temperatures, the rate constants for its adsorption, desorption, and dissociation have been estimated from the transition state theory (cf. Figure 2B). For all the temperatures explored, ranging 250–1000 K, CO_2_ desorption is faster than its dissociation on VC and NbC, suggesting that if adsorbed species are present, they will correspond to CO_2_ rather than CO and O. On the contrary, VC_1–*x*_ and TaC feature very similar rate constants for CO_2_ desorption and dissociation, so from the rate constants only, it is not clear what process will dominate. It is worth noting that, for TaC, the rate constant for CO_2_ desorption becomes faster than that of CO_2_ adsorption only above 617 K, which is a significantly higher temperature than that of VC_1–*x*_ (481 K) or NbC (494 K), making TaC a more suitable material for CCS technologies at high temperatures.

**Figure 3:**
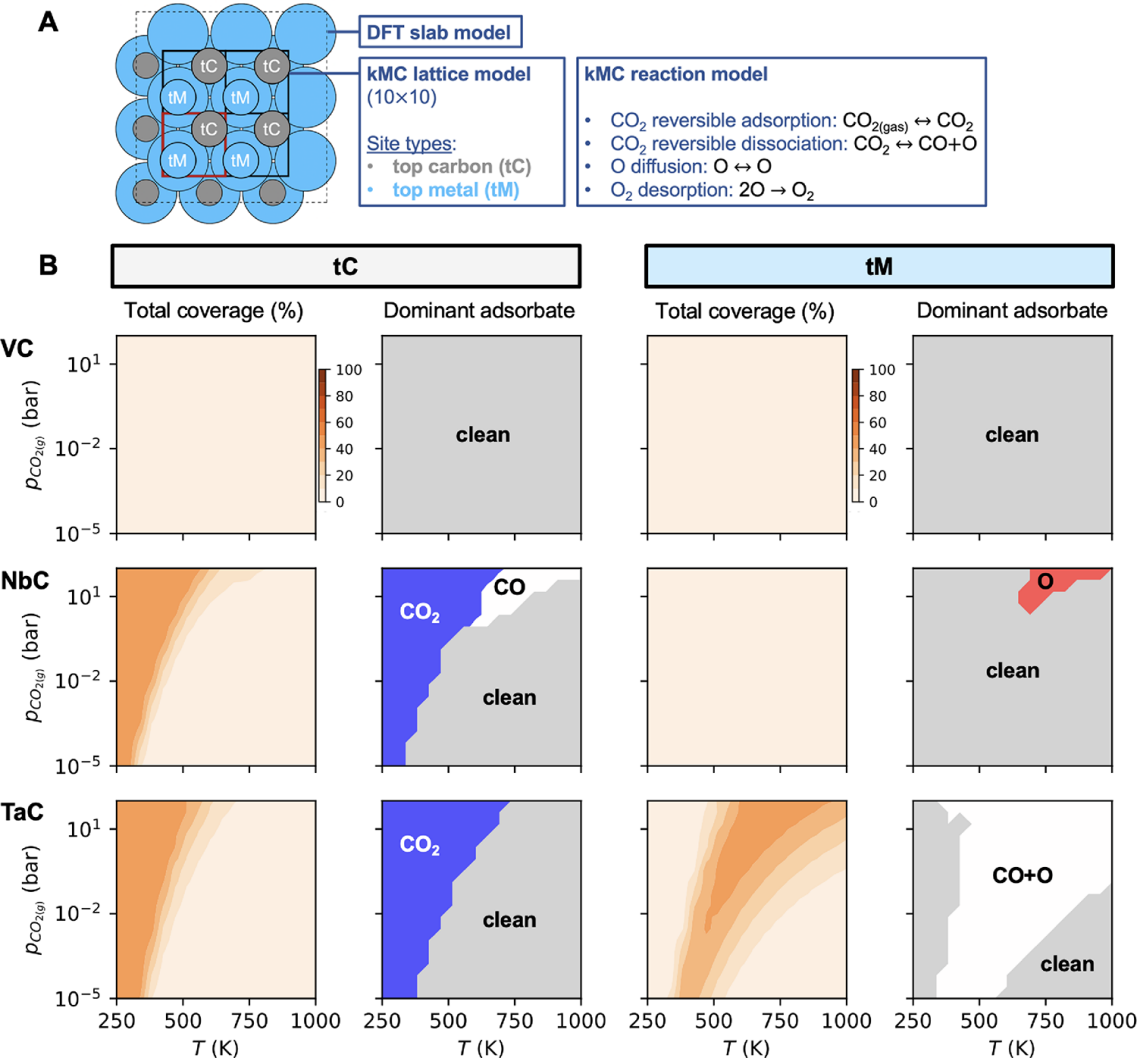
(A) Illustration of the DFT slab model and the kMC lattice model (superimposed) and list of the reactions included in the kMC reaction model. Blue and gray spheres represent metal and carbon atoms, respectively. (B) Total coverage and phase diagrams obtained from the kMC simulations as a function of CO_2_ partial pressure (10^–5^–10^2^ bar) and temperature (250–1000 K) for the (001) surfaces of VC, NbC, and TaC. In the phase diagrams, the most dominant species are only shown if the total coverage is higher than 5%. A total of 3 × 10^7^ and 7 × 10^7^ kMC steps are performed for equilibration and production, respectively, in all kMC simulations.

To shed some light on the nature of adsorbed species on the TMC surfaces for a wide range of temperatures and partial pressures, we have performed kMC simulations of CO_2_ adsorption and dissociation including the effect of lateral interactions on the stability of adsorbed species and energy barriers as well as diffusion of atomic O species (cf. Figure 3A). The total coverage and the corresponding phase diagrams at 250–1000 K and 10^–5^–10^2^ bar for VC, NbC, and TaC are shown in Figure 3B. Note that we have not performed kMC simulations on the VC_1–*x*_ model due to the complexities that arise from a dynamic lattice model in which the number of surface vacancies changes with time. The simulated phase diagrams agree very well with the predictions from the energy profiles and rate constants in Figure 2. For VC, the surface is always empty even at the lowest temperature (250 K) and highest pressure (100 bar) as a result of its very weak interaction with CO_2_. Therefore, the presence of adsorbed CO_2_ and CO species detected experimentally must be due to C vacancies. Regarding NbC, the kMC simulations indicate that the surface would be covered by CO_2_ at relatively low temperatures (<300–650 K depending on ) and clean in the remaining conditions, with the exception of high temperatures and very high pressures (>10 bar) where the most dominant species are CO and O. Finally, in the case of TaC, the kMC simulations indicate the presence of adsorbed CO_2_ species up to 350–700 K (depending on ) and CO + O at higher temperatures. Only in the case of high temperatures and very low  (<10^–2^ bar), a clean surface can be expected. A similar conclusion is expected for the case of VC_1–*x*_ because the potential energy diagrams are quite alike. Therefore, it is clear that the predicted CO_2_ adsorptive capabilities from the kMC simulations increase in the VC < NbC < TaC (≈ VC_1–*x*_) sequence, even to the point that TaC (and VC_1–*x*_) allow the CO_2_ capture and dissociation at near ambient conditions.

### 3.5 CO_2_ Reactivity Experiments

In addition to the calorimetric adsorption studies of CO_2_, the samples were subjected to CO_2_ flow at 873 K for 140 min and the released CO was quantified (see Table 1 and Figure S4 in the SI). The formation of CO was observed in all cases. VC-Pr exhibited a higher CO production (2913 mmolCO kg_cat_^–1^) compared to VC-Cla (2112 mmolCO kg_cat_^–1^) and VC-Clb (1980 mmolCO kg_cat_^–1^), which is attributed to a higher presence of surface C vacancies on VC-Pr. On the other hand, NbC and TaC produced only 271 and 226 mmolCO kg_cat_^–1^, respectively. Notably, an initial significant reduction in CO production was observed within the first minutes of reaction in all cases (Figure S4 in the Supporting Information). The XRD patterns obtained after the CO_2_ reactivity experiments at 873 K revealed the formation of crystalline metal oxides in VC-Pr, VC-Cla, VC-Clb, and NbC (Figure S5). No peaks corresponding to crystalline TaO_*x*_ were detected in TaC. This observation could be attributed to the higher oxidation resistance exhibited by TaC compared to NbC and VC systems.^41,42^

### 3.6 Simulated IR spectra

**Figure 4:**
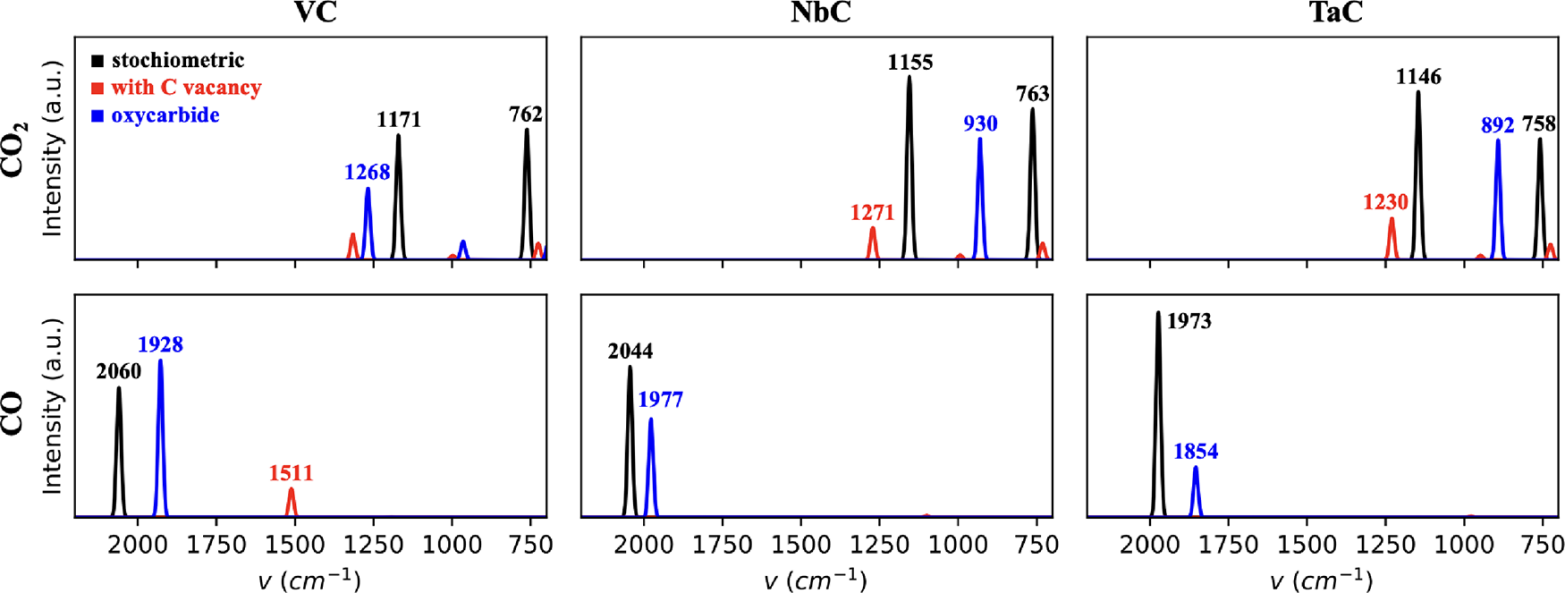
Simulated IR spectra in the range 2200–700 cm^–1^ of either adsorbed CO_2_ (top) and CO (bottom) or VC, NbC, and TaC (left to right). Main peaks are marked with their vibrational frequency. Black, red, and blue peaks correspond to the stoichiometric carbide, the carbide with a surface C vacancy, and the corresponding oxy-carbide.

To interpret the experimental spectra of the three Group 5 TMCs in the presence of CO_2_ and to identify the possible formation of CO or O species, we simulated the IR spectra of CO_2_ and CO species adsorbed on the three TMCs, as shown in Figure 4 and Table 2. According to the simulated spectra, molecular CO_2_ is characterized by the presence of two peaks at ∼760 cm^–1^ and 1146–1171 cm^–1^. The crucial factor here is that, upon dissociation, the resulting moieties would essentially lead to highly intense signals at 2044–2060 cm^–1^ for VC and NbC and ∼1973 cm^–1^ for TaC, which would correspond to the stretching vibration of a perpendicularly adsorbed CO molecule. Due to the possible presence of C vacancies and the possible oxidation of the outermost surface layer, we have also simulated the IR spectrum of adsorbed CO_2_ and CO on a surface vacancy site and on the corresponding oxy-carbide. The presence of surface C vacancies or an oxy-carbide phase can be detected from the IR spectra due to the appearance of new peaks at different frequencies (red and blue peaks in Figure 4). Noticeably, when CO is adsorbed on the corresponding oxy-carbide, the frequency of the C–O stretching peak is about 100 cm^–1^ lower compared to the stoichiometric TMC. The optimized geometries for all surface species on all TMC, TMC_1–*x*_, and TMOC slab models are displayed in Figures S6–S8 in the Supporting Information.

**Table 2:** Calculated IR Vibrational Modes (in cm^–1^) in the Range 2200–700 cm^–1^ for Adsorbed CO_2_ and CO Species on All TMC Surfaces Considered

**surface**	**CO**_**2**_	**CO**
VC	762: bending	2060: stretching
	1171: symmetric stretching	
VC_1–*x*_		1511: stretching
VOC	1268: asymmetric stretching	1928: stretching
NbC	763: bending	2044: stretching
	1155: symmetric stretching	
NbC_1–*x*_	1271: asymmetric stretching	
NbOC	930: symmetric stretching	1977: stretching
TaC	758: bending	1973: stretching
	1146: symmetric stretching	
TaC_1–*x*_	1230: asymmetric stretching	
TaOC	892: symmetric stretching	1854: stretching

### 3.7 Experimental IR spectra

#### 3.7.1 VC Samples

**Figure 5:**
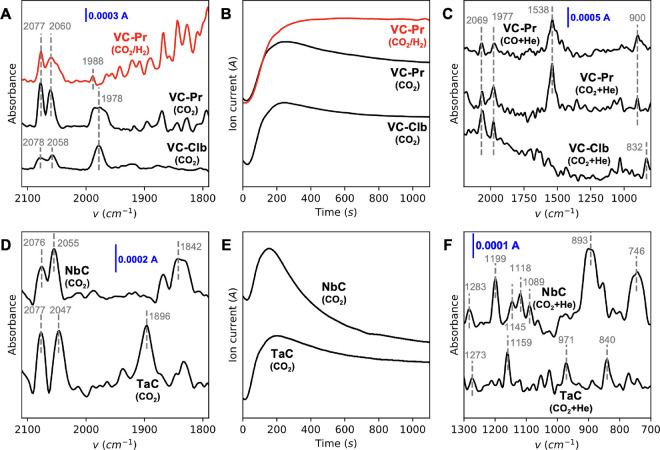
(A) Experimental DRIFT spectra after CO_2_ adsorption over VC-Pr and VC-Clb and after CO_2_/H_2_ adsorption over VC-Pr. (B) CO MS profile (*m*/*z* = 30) after CO_2_ adsorption over VC-Pr and VC-Clb and after CO_2_/H_2_ adsorption over VC-Pr. (C) Experimental DRIFT spectra after the adsorption of CO_2_ and He flushing over VC-Pr and VC-Clb and after CO adsorption and He flushing over VC-Pr. (D) Experimental DRIFT spectra after CO_2_ adsorption over NbC and TaC. (E) CO MS profile (*m*/*z* = 30) after CO_2_ adsorption over NbC and TaC. (F) Experimental DRIFT spectra after the adsorption of CO_2_ and He flushing over NbC and TaC; *T* = 308 K.

Figure 5A (black lines) shows the experimental DRIFT spectra for the adsorption of CO_2_ on VC-Pr and VC-Clb samples in the range 2100–1800 cm^–1^, where the stretching vibrations associated with coordinated CO are anticipated. VC-Clb was selected instead of VC-Cla due to a higher predominance of the stoichiometric VC phase. The two peaks observed at 2078–2058 cm^–1^ are in perfect agreement with the C–O stretching vibrations for adsorbed CO on VC predicted by DFT (Table 2). Meanwhile, the band at a lower wavenumber (1978 cm^–1^) can be attributed to the C–O stretching of CO on VOC. This suggests that both VC-Pr and VC-Clb samples expose VC and VOC phases, in agreement with the Raman bands in Figure 1C. Furthermore, the release of CO from the CO_2_-DRIFT experiments was monitored via MS analysis, as shown in Figure 5B (black lines). Upon contact of CO_2_ with the samples, the presence of CO was detected within the first 200 s, after which it gradually decreased over time in both samples. This phenomenon may be linked to the diminution of active sites responsible for CO_2_ dissociation (i.e., C vacancies), which are continuously converted into VOC. Notably, this decrease in CO production over time aligns well with the observations made when a CO_2_ stream was passed through the TMCs in the reactor (Figure S4).

After approximately 30 min, the CO_2_ flow was switched to He to remove the potential physisorbed species. The resulting DRIFT spectra for VC-Pr and VC-Clb, recorded in the wavenumber range of 2200–800 cm^–1^, are depicted in Figure 5C. The previously mentioned bands, now centered at 2069 and 1977 cm^–1^, remain visible, indicating the presence of chemisorbed CO species. Additionally, a well-defined band at 1538 cm^–1^ is observed in the spectrum of VC-Pr but is absent in the spectrum of VC-Clb. According to simulated IR calculations, this band can be attributed to the presence of surface C vacancies (C–O stretching on the VC_1–*x*_ model, see Table 2), in perfect agreement with the higher concentration of C vacancies of VC-Pr compared to VC-Clb. To validate the attribution of the aforementioned IR bands to different coordinated CO species, a separate CO adsorption experiment was conducted for the VC-Pr sample. Following CO adsorption and subsequent He flushing, the *in situ* DRIFT spectrum, presented in Figure 5C, displays bands at 2069, 1977, and 1538 cm^–1^, consistent with those observed previously.

In a separate experiment, the adsorption of a mixture of CO_2_/H_2_ = 1/3, simulating conditions of the RWGS reaction, was conducted over the VC-Pr sample. The DRIFT spectrum presented in Figure 5A (red line) exhibits similar yet broader bands in the range of 2077–2060 cm^–1^ under the CO_2_/H_2_ flow. However, a less intense band at 1988 cm^–1^ was observed in this case. As discussed previously, the band at 1988 cm^–1^ is likely attributed to the C–O stretching of CO over surface oxy-carbide species (cf. Table 2). The use of the CO_2_/H_2_ mixture instead of pure CO_2_ flow may facilitate the continuous conversion of VOC species on the surface by removing surface oxygen species with H_2_. Under these conditions, the online MS analysis indicates a continuous production of CO (red line in Figure 5B).

#### 3.7.2 NbC and TaC samples

Figure 5D displays the recorded CO_2_-DRIFT spectra for the NbC and TaC samples. Upon CO_2_ adsorption on the NbC sample, bands observed in the range of 2076–2055 cm^–1^, along with a broad absorption peak centered at 1842 cm^–1^, can be associated with coordinated CO on stoichiometric NbC and NbOC species, respectively (cf. Table 2). Similarly, for the TaC sample, bands in the range 2077–2047 cm^–1^ and a peak at 1896 cm^–1^ are indicative of coordinated CO on stoichiometric TaC and TaOC species, respectively (Table 2). The positions of these bands align quite well with those obtained from the simulated IR spectra (Figure 4).

The release of CO from CO_2_ adsorption on NbC and TaC samples was also monitored using online MS analysis (cf. Figure 5E). CO was detected within the first 200 s of CO_2_ adsorption, after which the evolution of the CO gradually decreased with time on both samples. Furthermore, after approximately 30 min, the flow of CO_2_ was switched to He to remove physisorbed species. Figure 5F illustrates the CO_2_-DRIFT spectra of NbC and TaC after He flushing, within the wavenumber range 1300–700 cm^–1^. For NbC, the dominant presence of a stoichiometric NbC phase is indicated by the bands at 746 cm^–1^ (CO_2_ bending) and in the range 1089–1199 cm^–1^ (CO_2_ stretching), according to Table 2. The intense band observed at 893 cm^–1^ indicates the presence of an oxy-carbide phase (CO_2_ stretching on NbOC). The small band at 1283 cm^–1^ might indicate the presence of some surface C vacancies. Regarding TaC, the presence of a stoichiometric TaC phase is indicated by the strong band at 1159 cm^–1^ (CO_2_ stretching). The bands at 840 and 971 cm^–1^ could be attributed to the CO_2_ stretching on TaOC. Finally, the very small band at 1273 cm^–1^ could indicate the presence of some surface C vacancies (CO_2_ stretching on TaC_1–*x*_). Thus, the presence of C vacancies cannot be ruled out in NbC and TaC samples, although its concentration is expected to be much lower compared to that in the VC samples, because the intensities of the bands at 1283 and 1273 cm^–1^ for NbC and TaC are at least 10 times lower than that of the band at 1538 cm^–1^ for VC-Pr (see Figure 5C,F).

The detection of adsorbed CO_2_ in the CO_2_-DRIFT spectra of NbC and TaC following He flushing suggests a stronger adsorption of molecular CO_2_ on NbC and TaC compared to that of VC samples. This finding is consistent with the CO_2_ adsorption heat values obtained in TGA experiments (refer to Table 1), where NbC and TaC displayed higher CO_2_ adsorption heat values compared to those of VC samples. Moreover, DRIFTS results confirmed the coexistence of stoichiometric and oxy-carbidic surfaces in both NbC and TaC samples and suggested the possible presence of surface C vacancies, although to a very minor extent compared to the VC samples.

### 3.8 RWGS Catalytic Tests

**Figure 6:**
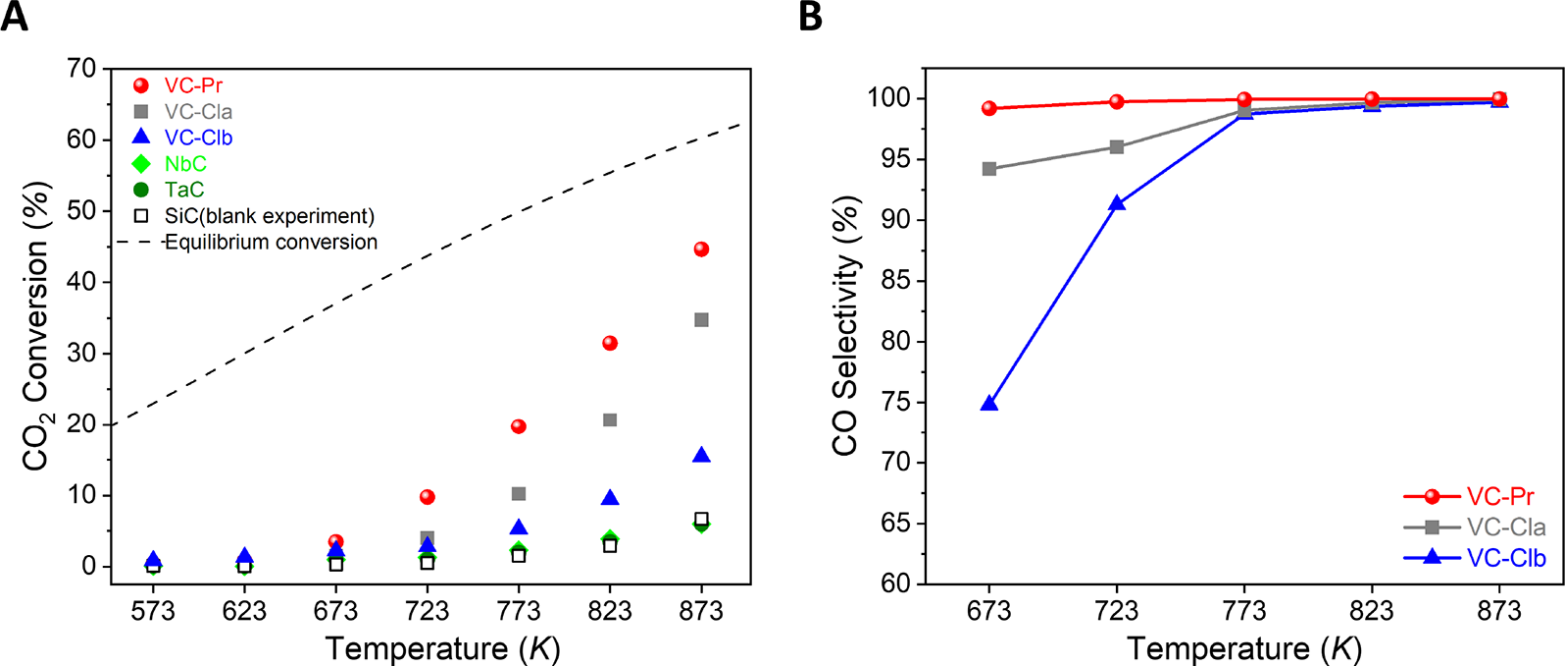
Catalytic behavior of the Group 5 TMCs in the RWGS reaction as a function of reaction temperature: (A) CO_2_ conversion and (B) CO selectivity. Reaction conditions: *m*_cat_ = 150 mg, CO_2_/H_2_/N_2_ = 1/3/1, GHSV = 3000 h^–1^, *p* = 1 bar.

Based on the above experiments, it becomes apparent that all of the considered samples can capture and activate CO_2_ at room temperature. Notably, in the case of VC samples, this ability stems exclusively from the presence of surface carbon vacancies. Consequently, we investigated the potential use of such carbides as CO_2_ hydrogenation catalysts by means of the RWGS reaction. As depicted in Figure 6, only the VC samples among all the tested materials are active toward the RWGS. The observed trend in CO_2_ conversion and CO selectivity—VC-Pr > VC-Cla > VC-Clb—is closely tied to the concentration of highly active surface C vacancy sites. Conversely, NbC and TaC exhibit poor catalytic activity. In fact, the poor performance of the stoichiometric Group 5 TMCs was predicted in a previous DFT study,^43^ given their very weak interaction with H_2_ and the endoergonicity of the H_2_ dissociation reaction. However, the presence of surface C vacancies allows for a much stronger binding of H species, i.e., the adsorption energies for adsorbed H species on VC and VC_1–*x*_ are −2.03 and −2.91 eV, respectively,^15^ and also facilitates H_2_ dissociation, given that the potential energy barriers for H_2_ dissociation on VC and VC_1–*x*_ are 0.65 and 0.16 eV, respectively.^15^ Therefore, our results validated these theoretical predictions, and the poor RWGS activity of NbC and TaC can be attributed to their feeble interaction with adsorbed H_2_ or H species and presumably also to their limited concentration of C vacancies.

## 4 Conclusions

The present study evaluates the potential of VC, NbC, and TaC as materials for CCS and CCU technologies. In the case of VC, the C-defective V_8_C_7_ phase is more stable than the stoichiometric (cubic) VC phase, and three different samples with varying concentrations of C vacancies are prepared and examined. This is not the case for NbC and TaC, where the stoichiometric phase is predominant. Calorimetric measurements of adsorbed CO_2_ align closely with the present PBE-D3 calculations on CO_2_ adsorption, validating the computational methodology. Subsequently, free energy diagrams for CO_2_ dissociation and kMC simulations are employed to predict the surface composition of VC, NbC, and TaC under different conditions of  and *T*, leading to the construction of phase diagrams. This information is compared with experimental DRIFT spectra after CO_2_ adsorption. To interpret the experimental IR spectra, we compute the interaction of CO_2_, CO, and O with DFT not only on the stoichiometric TMC models but also on slab models with a surface C vacancy (TMC_1–*x*_) and their corresponding oxy-carbides (TMOC). Then, we simulate the IR spectra through vibrational analysis and use this information to reveal the presence of C vacancies and the oxy-carbide phase on the different samples.

Our findings reveal that VC is a promising material for CCU owing to the critical role played by surface C vacancies, which are found in abundance in this material. Without these vacancies, VC would weakly interact with CO_2_, much less dissociate it. Conversely, NbC and TaC show potential for CCS due to their strong interaction with CO_2_, and in the case of TaC, it can easily dissociate CO_2_ to CO + O at a low temperature. However, both NbC and TaC show poor intrinsic catalytic performance for CO_2_ hydrogenation *per se*, and we attribute this result to the much lower concentration of C vacancies present in these carbides. The present catalytic tests have also validated earlier theoretical predictions suggesting that the stoichiometric phases of Group 5 TMCs exhibit poor performance in the hydrogenation of CO_2_.^42^ We propose that the catalytic activity for NbC and TaC could also be improved by the incorporation of C vacancies through novel preparation methods, or using promoters, such as small metal clusters.^8,44^ This study advocates for the use of Group 5 TMCs in CCS or CCU technologies, offers a guide on utilizing IR spectra as a fingerprint for identifying oxy-carbide phases or surface C vacancies within Group 5 TMCs, and highlights the importance of surface C vacancies in the adsorptive and catalytic properties of TMCs.

## Supporting Information Available

Additional details on the experimental and theoretical techniques, plots of CO production from CO_2_ reactivity studies, XRD patterns of samples after CO_2_ reactivity, illustrations of all adsorbed configurations on TMC, TMC_1–*x*_, and TMOC slab models (TM = V, Nb, and Ta), and calculated vacancy formation energies (PDF)
