# Supplementary material for: On the Capabilities of Transition Metal Carbides for Carbon Capture and Utilization Technologies
Source: ACS Appl Mater Interfaces. 2024 May 24;16(22):28505–16. doi: 10.1021/acsami.4c03735 (PMC11163407; doi:10.1021/acsami.4c03735)

On the Capabilities of Transition Metal Carbides for Carbon Capture and Utilisation Technologies

Hector Prats,[a,¶,*] Arturo Pajares,[b,¶] Francesc Viñes,[c] Pilar Ramírez de la Piscina,[d] Ramon Sayós,[c] Narcís Homs,[d,e,*] and Francesc Illas[c]

[a] *Department of Chemistry, Physical & Theoretical Chemistry Laboratory, University of Oxford, South Parks Road, Oxford OX1 3QZ, UK*

[b] *Sustainable Materials, Flemish Institute for Technological Research (VITO NV), Boeretang 200, 2400 Mol, Belgium.*

[c] *Departament de Ciència de Materials i Química Física & Institut de Química Teòrica i Computacional (IQTCUB), Universitat de Barcelona, Martí i Franquès 1-11, 08028 Barcelona, Spain.*

[d] *Departament de Química Inorgànica i Orgànica, Secció de Química Inorgànica and Institut de Nanociència i Nanotecnologia (IN2UB), Universitat de Barcelona, Martí i Franquès 1-11, Barcelona, 08028, Spain.*

[e] *Institut de Recerca en Energia de Catalunya (IREC), Jardins de les Dones de Negre 1, Barcelona, 08930, Spain.*

Corresponding authors: Narcís Homs (narcis.homs@qi.ub.es) and Hector Prats (hector.pratsgarcia@chem.ox.ac.uk)

[¶] Both authors contributed equally

Contents: **Page**

Section	Page
Section S1: Experimental details	S2
Section S2. Experimental results	S3
Section S3. Computational details	S8
Section S4. Computational results	S9

Section S1. Experimental details

Amount of the metal and C precursors:

The amount of the metal and C precursors added for the preparation of the different samples is listed below.

- VC-Pr: 14.29 mmol of VO(isopropoxide)$_3$ and 19.29 mmol of 4,5-dicyanoimidazole.
- VC-Cla: 14.29 mmol of $VOCl_3$ and 14.89 mmol of 4,5-dicyanoimidazole.
- NbC: 9.5 mmol of $Nb(OC_2H_5)_5$ and 9.9 mmol of 4,5-dicyanoimidazole.
- TaC: 4.66 mmol of $TaCl_5$ and 8.5 mmol of 4,5-diciyanoimidazole.

Section S2: Experimental results

Figure S1. TEM images of as-synthesized carbides.

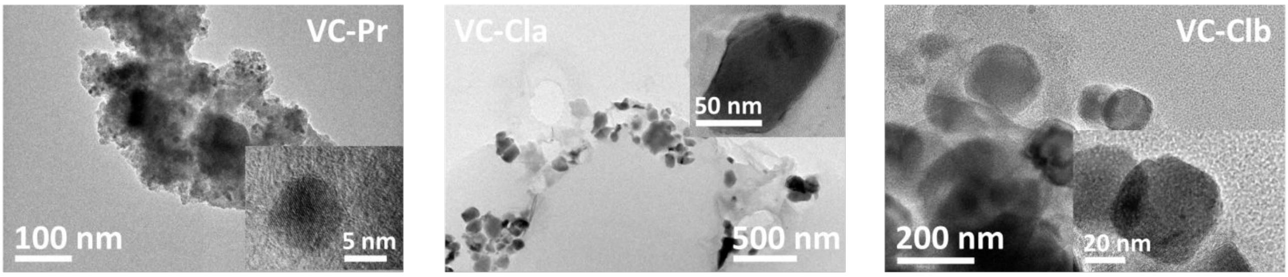

Figure S2. SEM images of as-synthesized carbides**.**

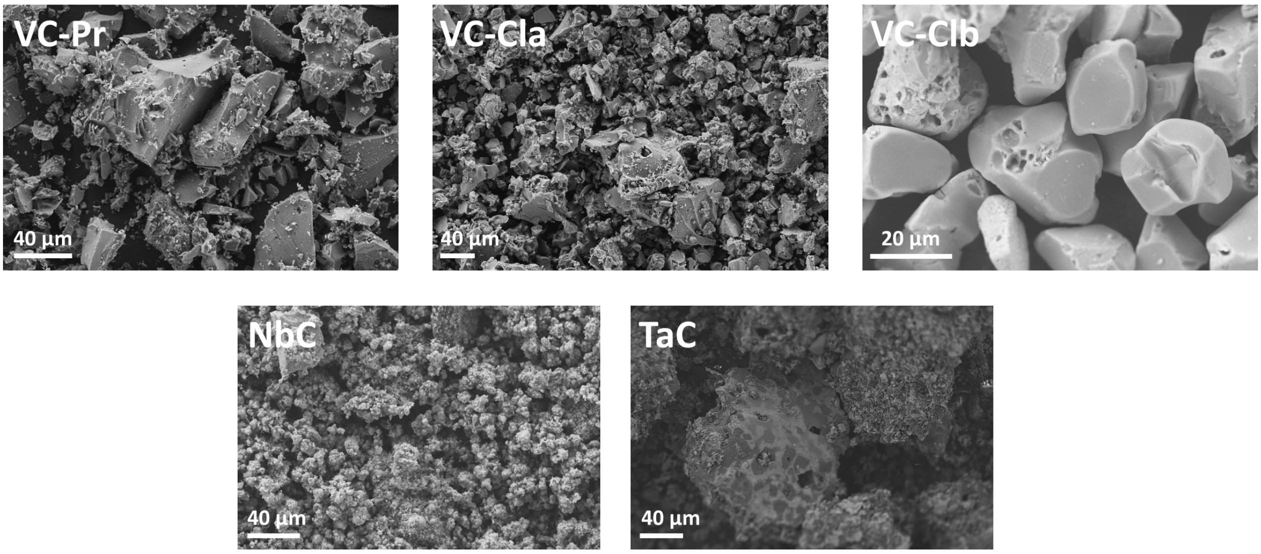

Figure S3. N_2 adsorption-desorption isotherms and pore size distribution (inset) of as-synthesized carbides.

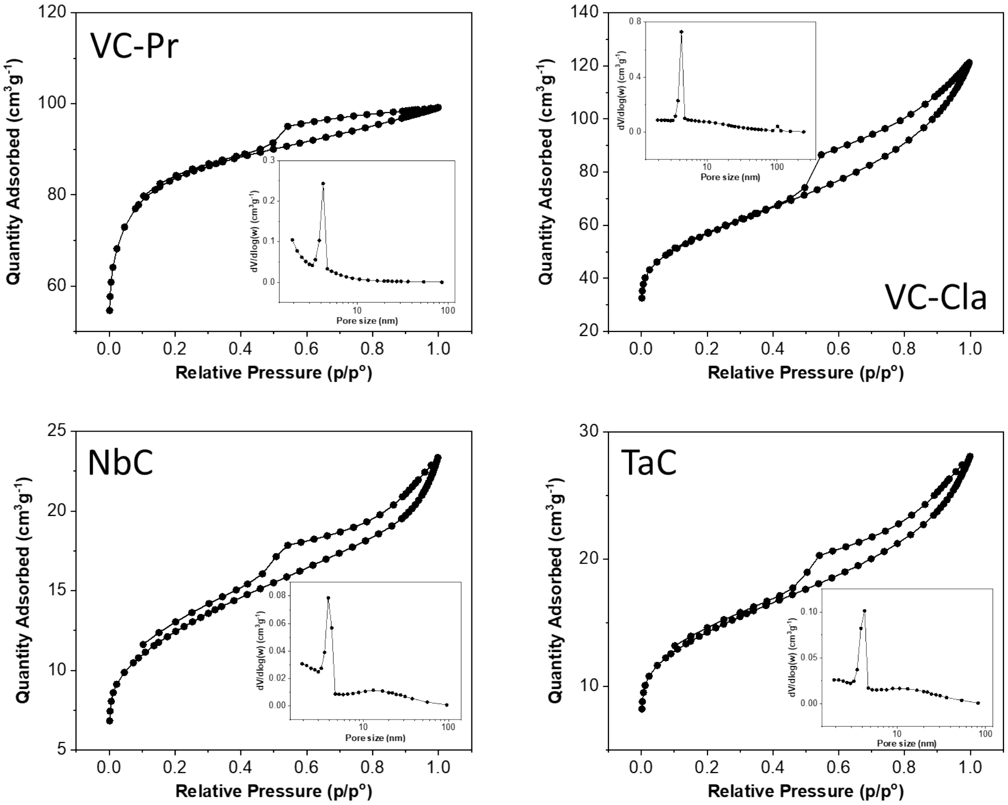

Figure S4. CO production from CO_2 reactivity studies on samples. Reaction conditions: m_{cat} = 150 mg, CO_2/Ar = 1/5, GHSV = 3000 h^{-1}, p = 1 bar, T = 873 K.

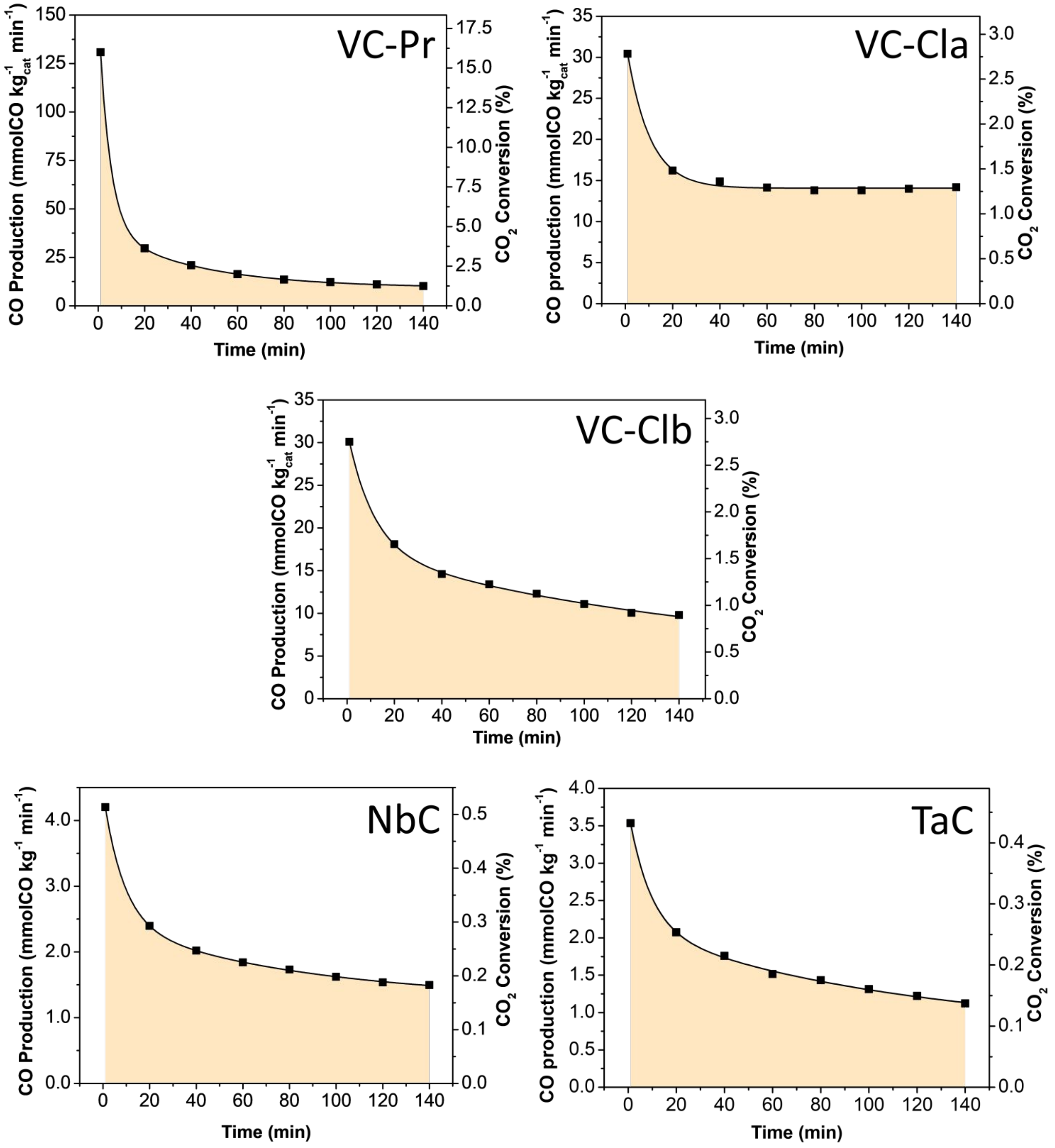

Figure S5. XRD patterns of samples after CO_2 reactivity. Reaction conditions: m_{cat} = 150 mg, CO_2/Ar = 1/5, GHSV = 3000 h^{-1}, p = 1 bar, T = 873 K.

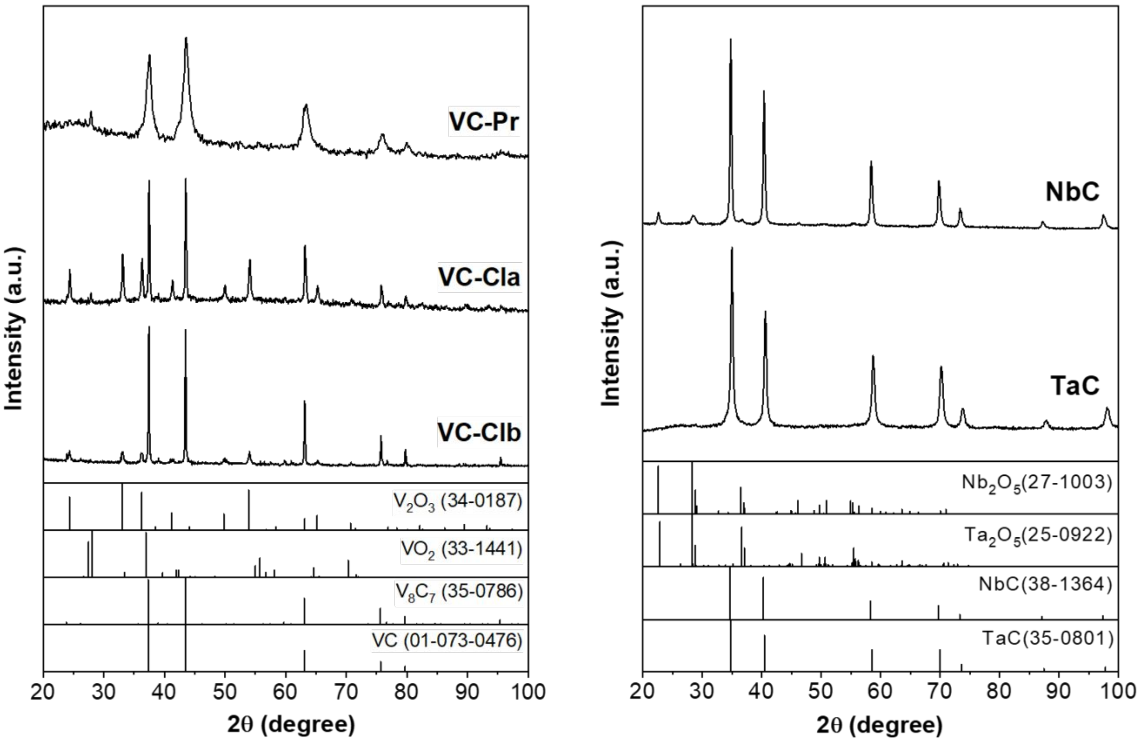

Section S3: Computational details

The formation energies for CO_2, CO, and O species ($E_{f,i}$) are calculated with respect to gas-phase CO_2 and O_2 molecules:

$$E_{f,CO_2} = E_{CO_2+slab} - E_{slab} - E_{CO_2(g)} \quad \text{(S1)},$$

$$E_{f,CO} = E_{CO+slab} - E_{slab} - E_{CO_2(g)} - \frac{1}{2}E_{O_2(g)} \quad \text{(S2)},$$

$$E_{f,O_2} = E_{O_2+slab} - E_{slab} - \frac{1}{2}E_{O_2(g)} \quad \text{(S3)},$$

where $E_{i,slab}$ is the energy of the species i adsorbed on the corresponding slab model (VC, NbC, or TaC), E_{slab} is the energy of the relaxed pristine slab, and $E_{i(g)}$ is the energy of the species i in gas-phase, calculated in an asymmetric box of 9×10×11 Å and at **Γ**-point.

Similarly, the formation energy of a C vacancy, $E_{f,vac}$, is calculated as follows:

$$E_{f,vac} = E_{slab-vac} + E_C - E_{slab} \quad \text{(S4)},$$

where $E_{slab-vac}$ is the energy of the relaxed slab with a surface C vacancy, and E_C is the energy of a C atom in the bulk of its most stable phase (graphite), calculated as the total energy of a bulk unit cell of graphite divided by the total number of C atoms in the unit cell. Within these definitions, the more negative the formation energy is, the more stable is the adsorbed species or vacancy.

Section S4: Computational results

Table S1. Reaction models for the kinetic Monte Carlo (kMC) simulations.

**VC**
$CO_{2(g)} + site_{(tC)} \leftrightarrow CO_{2(tC)}$
$O_{2(g)} + 2site_{(tC)} \leftrightarrow 2O_{(tC)}$
$CO_{2(tC)} + site_{(tC)} \leftrightarrow CO_{(tC)} + O_{(tC)}$
$O_{(tC)} + site_{(tC)} \leftrightarrow site_{(tC)} + O_{(tC)}$
**NbC**
$CO_{2(g)} + site_{(tC)} \leftrightarrow CO_{2(tC)}$
$O_{2(g)} + 2site_{(tM)} \leftrightarrow 2O_{(tM)}$
$CO_{2(tC)} + site_{(tM)} \leftrightarrow CO_{(tC)} + O_{(tM)}$
$O_{(tM)} + site_{(tM)} \leftrightarrow site_{(tM)} + O_{(tM)}$
**TaC**
$CO_{2(g)} + site_{(tC)} \leftrightarrow CO_{2(tC)}$
$O_{2(g)} + 2site_{(tM)} \leftrightarrow 2O_{(tM)}$
$CO_{2(tC)} + 2site_{(tM)} \leftrightarrow site_{(tC)} + CO_{(tM)} + O_{(tM)}$
$O_{(tM)} + site_{(tM)} \leftrightarrow site_{(tM)} + O_{(tM)}$

Table S2. Cluster expansion terms used in the kMC simulations. All values are given in eV.

Name	VC	NbC	TaC
**CO_2_point**	-0.148	-0.930	-1.166
**CO_point**	2.122	1.951	2.009
**O_point**	-2.120	-2.762	-3.140
**CO_2+CO_2_pair**	0.236	0.343	0.414
**CO_2+CO_pair**	0.131	0.309	0.071
**CO_2+O_pair**	0.134	0.272	0.337
**CO+CO_pair**	0.111	0.376	0.035
**CO+O_pair**	0.459	0.403	0.065
**O+O_pair**	0.209	0.420	0.414

Table S3. Calculated vacancy formation energies, $\boldsymbol{E_{f,vac}}$, for a surface C vacancy on different TMC (001) surface slab models. Values are given in eV.

**Slab**	$\boldsymbol{E_{f,vac}}$
**VC (001)**	-0.26
**NbC (001)**	-0.10
**TaC (001)**	0.23

Figure S6. Top views of all adsorbate configurations on VC slabs. Colour code of the spheres: C – dark grey, V – light grey, and O – red.

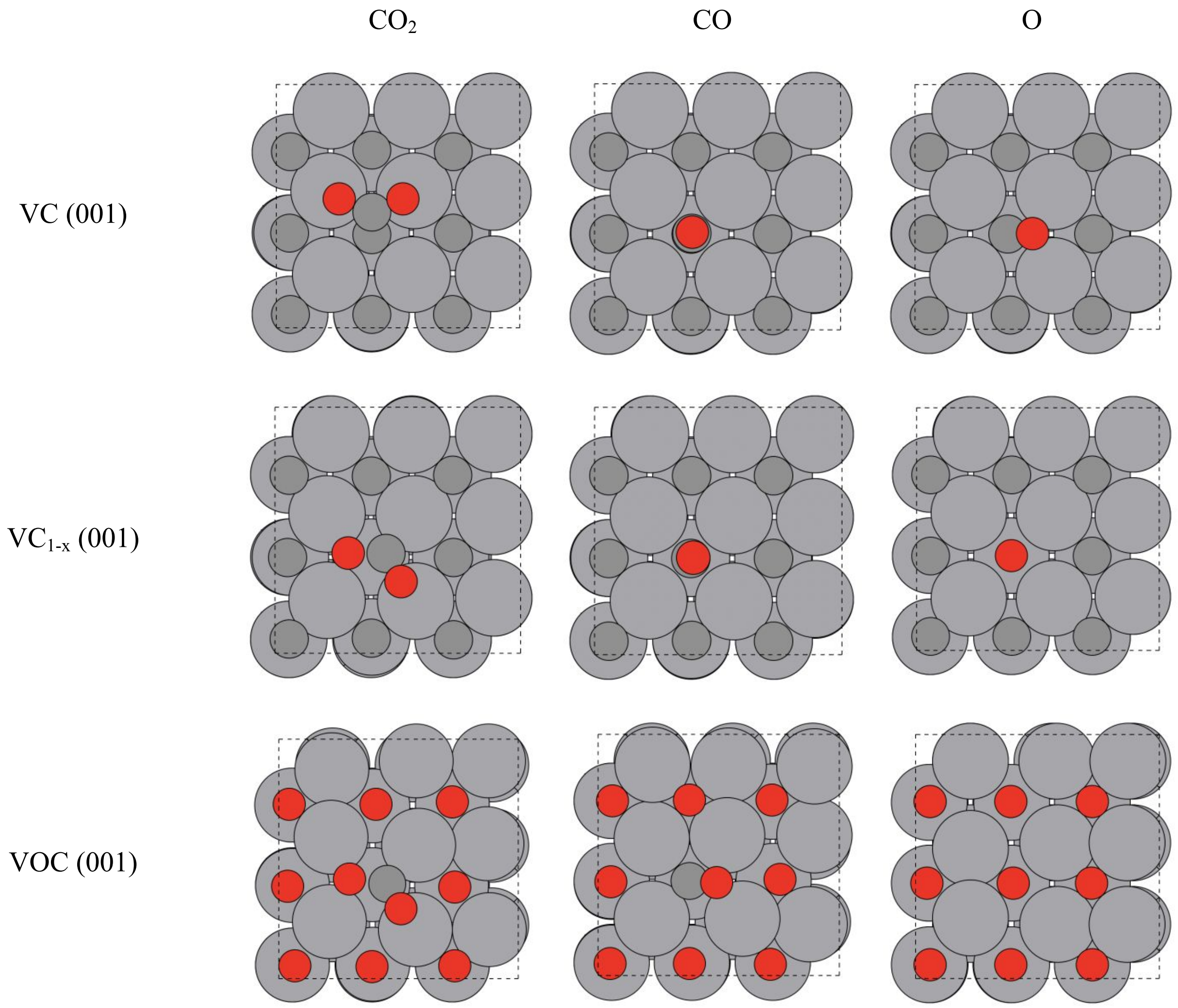

Figure S7. Top views of all adsorbate configurations on NbC slabs. Colour code of the spheres: C – dark grey, Nb – green, and O – red.

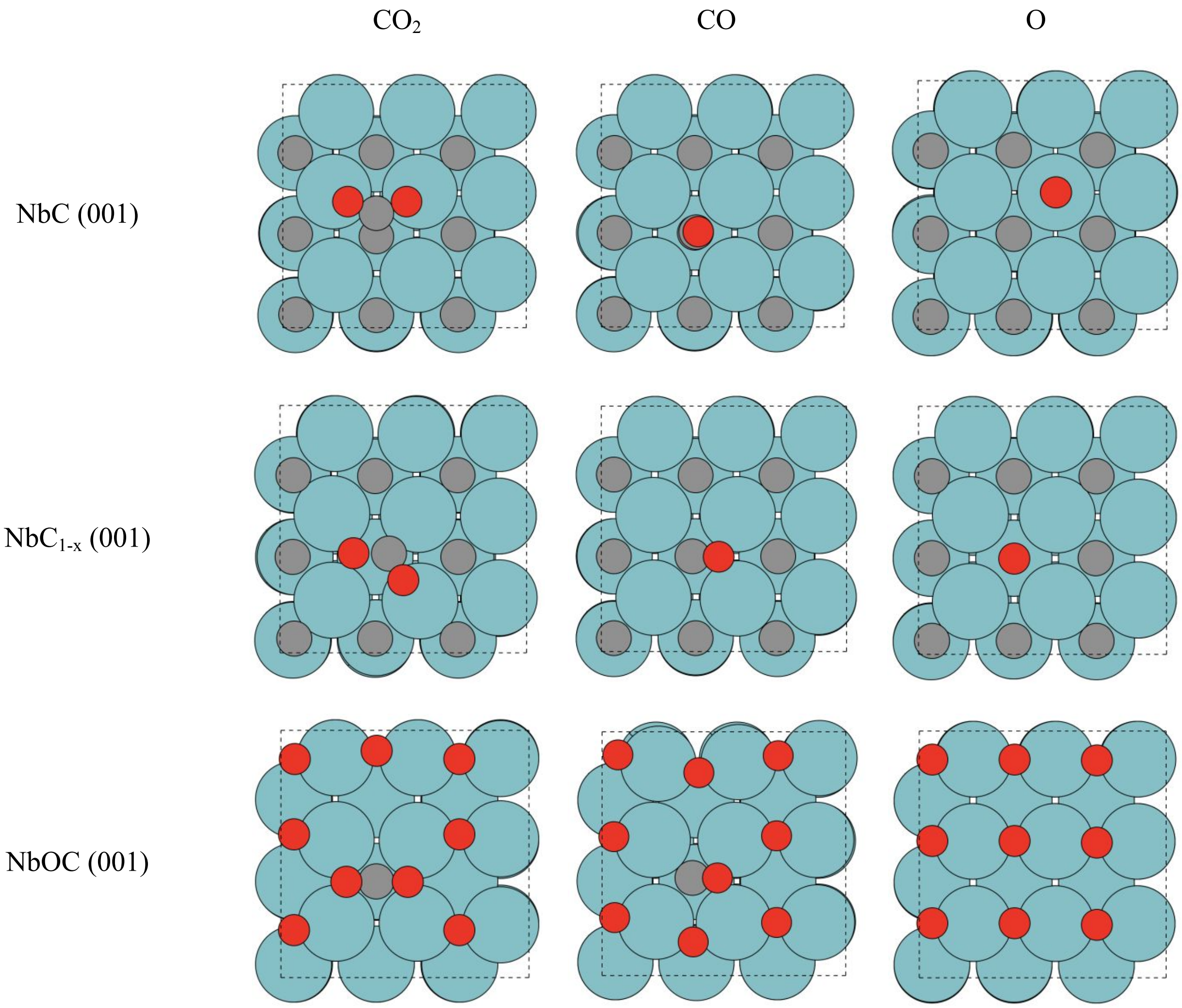

Figure S8. Top views of all adsorbate configurations on TaC slabs. Colour code of the spheres: C – dark grey, Ta – blue, and O – red.

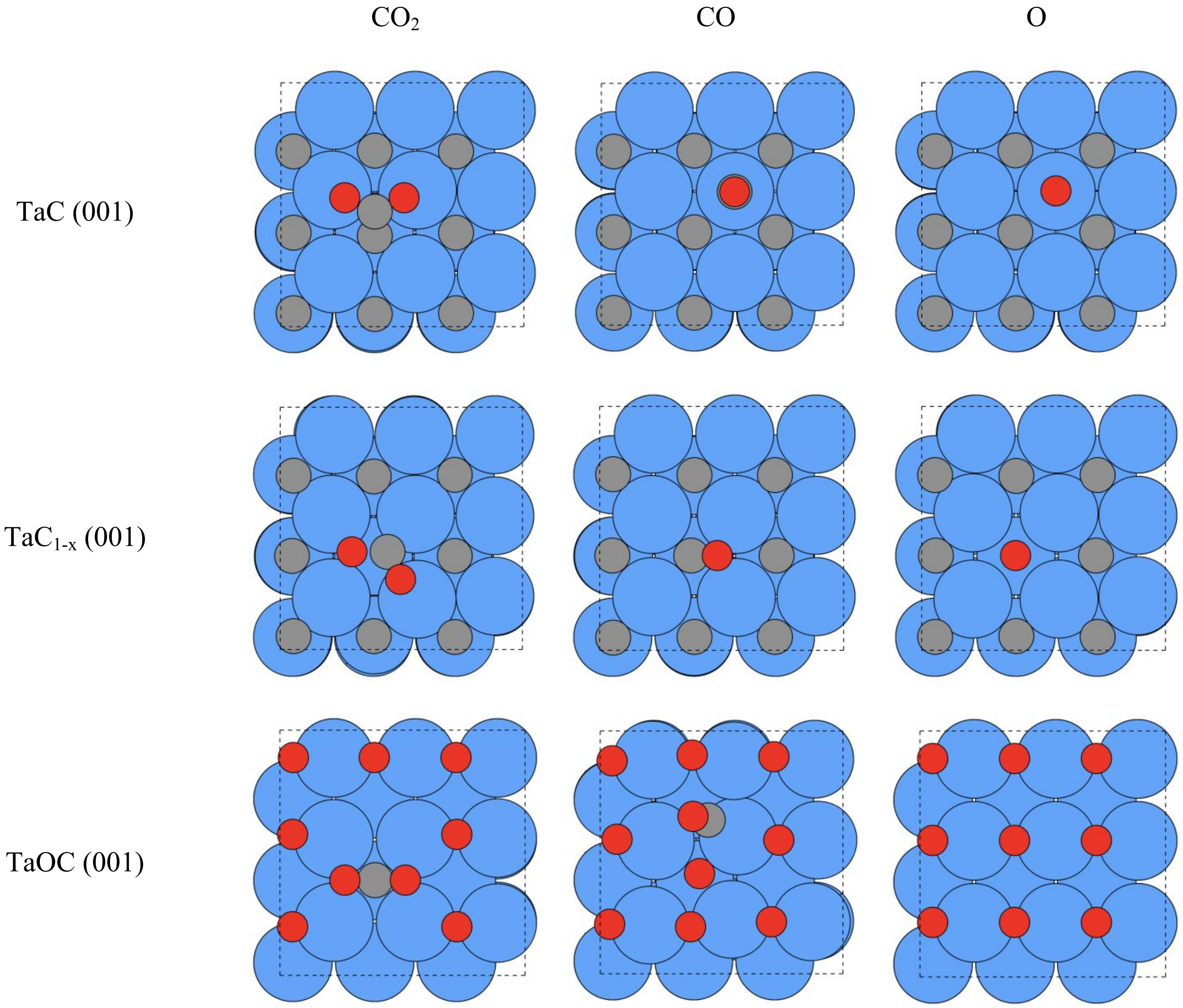

Supplement: Supplementary file 1 — am4c03735_si_001.pdf [file am4c03735_si_001.pdf]
